# Important Letter from the Chairman, with the Report of the Medical Board

**Published:** 1918-04-20

**Authors:** 


### ESSEX COUNTY HOSPITAL.

Our readers will remember that in our issue of the 30th ultimo, page 557, we published the substance of a letter which purported to give an account of the alleged state of affairs at the Essex County Hospital, Colchester. We ventured to conclude at the outset that neither the committee nor the medical staff could realise the possibility of such a condition of affairs, as described, existing in any institution for which they were in whole or in part responsible. We have now had an opportunity of going into the facts and the position as they are to-day, with the result, as the communications from the chairman and the medical staff demonstrate, that the alleged state of affairs described is confuted by the evidence given. We have great pleasure in publishing the refutation of the chairman of the hospital, Mr. H. B. Dickinson, J.P., and of Dr. H. B. Nicholson, the chairman of the Medical Board, and Dr. Henry Rowland, its honorary secretary. The initial difficulties arose when the matron of this hospital, like so many others, was called up, in October 1914, for service. She is now on war duty in Salonika, whence her resignation was recently sent. The difficulties created by the loss of a valued matron at the outset of the war, and what they have resulted in, are fully set out by Mr. Dickinson, and his letter, in conjunction with the report of the Medical Board, disposes of the statement of our correspondent and vindicates the hospital authorities. This vindication is supported by other evidence. We welcome the frank and thorough manner in which Mr. H. B. Dickinson, the new chairman, and the honorary medical staff have stated the position and justified the hospital administration and those responsible for it. Hospital opinion will welcome the grateful recognition by the staff of the excellent medical service rendered by the two ladies who, much to their credit, came to the rescue of the hospital at a time when it was almost impossible to fill the vacancies in resident hospital appointments. The staff's letter contains what, we are assured, is a well-merited and valuable testimony, not only to these two ladies' efficiency, but to their skill and devotion in the discharge of their arduous duties. The hospital has now the good fortune to have in Miss Mary E. Jones a matron of experience and character. Her special knowledge and ability are guarantees that the training-school under her direction will be found by probationers to be characterised by the thoroughness and excellence of its teaching, and the comfort and advancement of all who may train at the Essex County Hospital. Miss Jones entered on her duties on March 1, and in response to the chairman's expressed wish we shall have great pleasure in inspecting the institution a little later on.—Editor, The Hospital.

### Important Letter from the Chairman, with the Report of the Medical Board.

*To the Editor of* The Hospital.

Sir,—The duty of replying to your article [*i.e.* the letter] *re* this hospital published March 30, 1918, devolves upon me in consequence of the recent decease of the late Rev. Edward Spurrier, chairman of the committee for the last seven years and for thirty years a member of it. I am thankful he was spared at the end of a long life of good work for his fellows the bitter pain of reading such slanderous and untruthful accusations about the institution he had loved so well and ably controlled for so many years.

The substance of the letter which you feel it your duty to publish, you say more or less obliges you to conclude "that neither the committee nor the medical staff can realise the possibility of such a condition of affairs existing in any institution for which they are in whole or in part responsible," and to this I would add, nor if correct could they file any plea of extenuating circumstances. If your correspondent is in a position to know the facts, as you say he or she ought to be, it is difficult to imagine the state of mind which could account for such perversion.

Up to the commencement of the war you say the hospital had a good name. Only those who have had opportunities of seeing and visiting it realise how much this has been enhanced in consequence of the services rendered to the country since that period, under difficulties no doubt experienced by all institutions of similar character, by enlarging its accommodation to its fullest capability for the reception of our wounded soldiers direct from the battlefields of France.

Its matron was called up in October 1914 for service, but the senior sister, who remained as acting matron, was an excellent choice, a most able, sympathetic, and capable woman, respected and loved by all who knew her. As the strain of work was telling on her physical powers, a lady who had done good work until then as Commandant of a Red Cross detachment (and in connection with whom it would be unnecessary to say anything in the environment or precincts of the hospital, where her work since this war began, until now, is too well known for anything to her detriment to be believed) was persuaded to come and help her with the military work, and in any and every way possible assist her in work which did not require professional training. This occurred in September 1915. When the acting matron went away ill in March 1917, a senior sister was appointed temporarily to undertake the professional duties as sister-in-charge during her absence, and as soon as it was realised by her medical attendant's report that it was unlikely that she (the acting matron) could ever return, the Lady Commandant alluded to was most anxious and strongly urged that a properly qualified matron should be appointed, unless the matron who had been called up should, under the circumstances, be able to obtain release from military service.

Letters were sent to the original matron, then serving in Salonika, and to ensure receipt copies were posted at intervals of a few days to inquire as to the possibility of her being able to obtain release from military service to return to her original charge. Her answer when received, "given much against her personal desire to eventually return here," left her resignation in the hands of the committee, as she realised the difficulty of obtaining the services of a really efficient woman as temporary matron.

[Here follow certain personal statistics which we are advised not to publish.]

The resident staff, two Indian ladies, trained at the Royal Free Hospital, London, who are infamously libelled by your correspondent, have done most valuable service, and worked without sparing themselves night or day, early and late, and the report which I have requested the hon. medical staff to send me, with their collective judgment on the statements of your correspondent, should satisfy you of this, and as to allegations of non-enforcement of strict asepsis will lead you to a different conclusion as to where such a charge should lie.

In conclusion, Sir, I request that you or some other leading hospital authority should make an immediate inspection of this institution, and on the spot institute any inquiries you think advisable, in order that you may be in a position to judge what condition of mind could possibly have inspired the patriotic spirit of your correspondent! Yours faithfully,

H. B. Dickinson, Chairman.

Essex County Hospital, Colchester.

April 15, 1918.

### The Medical Board's Report.

Dear Mr. Dickinson,—You have asked for an expression of opinion by the members of the medical board on the article appearing in The Hospital (30/3/18).

In order to reply to your request we at once convened a meeting of the honorary medical staff, and unanimously agreed to put before you the following facts :—

[Here follow certain personal statistics which we are advised not to publish.]

Referring to the statements in the article which you brought specially to our notice, it is true that five sisters happened to leave in three months. Two of these were called up for military service, having been for six months in the Q.A.I.M.N.S. Reserve. One left to take the position of home sister in a large military hospital, one had to go on account of ill-health, and the sister-in-charge left when the new matron arrived.

[Personal Statistics.]

We note with amazement and indignation the phrases "the absence of decorum" and "the exhibition of reverent regard of life in the operation theatre." Nothing that could conceivably justify the use of such words has come to our notice, directly or indirectly, and we totally repudiate the apparent imputation.

[Personal Statistics.]

With regard to the resident staff, we unanimously state that the two ladies (who, we are thankful to say, continue to render the hospital service) have proved themselves most competent and thoroughly up to all recent medical and surgical work. They are most tactful in all their dealings with the nursing and honorary staff, and with the patients, and have won the esteem of all. They are unfailing in their devotion to their very arduous duties.

Should it be decided to ask for an inquiry into these charges or the general efficiency of the hospital, we should not only welcome it, but be ready at any inconvenience to assist and give evidence.—Yours sincerely,

(Signed) H. B. Nicholson, M.B., C.M.,
*Chairman of the Medical Board.*
Penry Rowland, M.D.,
*Hon. Secretary.*

H. B. Dickinson, Esq., J.P.
Essex County Hospital, Colchester, April 11, 1918.

